# Objective evaluation of surgical competency for minimally invasive surgery with a collection of simple tests

**Published:** 2016-03-30

**Authors:** Eliana Maria Gonzalez-Neira, Claudia Patricia Jimenez-Mendoza, Daniel R Suarez, Saul Rugeles-Quintero

**Affiliations:** 1 Departamento de Ingeniería Industrial, Facultad de Ingeniería, Pontifica Universidad Javeriana, Bogotá, Colombia; 2 Departamento de Cirugía, Pontificia Universidad Javeriana, Hospital Universitario San Ignacio, Bogotá, Colombia

**Keywords:** Proficiency-based training, laparoscopic skill assessment, simulation

## Abstract

**Objective:**

This study aims at determining if a collection of 16 motor tests on a physical simulator can objectively discriminate and evaluate practitioners' competency level, i.e. novice, resident, and expert.

**Methods:**

An experimental design with three study groups (novice, resident, and expert) was developed to test the evaluation power of each of the 16 simple tests. An ANOVA and a Student Newman-Keuls (SNK) test were used to analyze results of each test to determine which of them can discriminate participants' competency level.

**Results:**

Four of the 16 tests used discriminated all of the three competency levels and 15 discriminated at least two of the three groups (α= 0.05). Moreover, other two tests differentiate beginners' level from intermediate, and other seven tests differentiate intermediate level from expert.

**Conclusion:**

The competency level of a practitioner of minimally invasive surgery can be evaluated by a specific collection of basic tests in a physical surgical simulator. Reduction of the number of tests needed to discriminate the competency level of surgeons can be the aim of future research.

## Abstract (es)

**Objetivo:**

Este estudio pretende determinar si una colección de 16 pruebas en un simulador físico puede discriminar y evaluar objetivamente el nivel de competencia de practicantes de cirugía laparoscópica (novato, resistente y experto).

**Métodos:**

Se realizó un diseño de experimentos con tres grupos de estudio (novatos, residentes y expertos) para probar el poder de discriminación de 16 pruebas simples. Un ANOVA y un test de Student Newman-Keuls (SNK) fueron empleados para analizar los resultados de cada prueba con el fin de determinar cuáles de ellas podían discriminar los niveles de competencia de los participantes.

**Resultados:**

Cuatro de las 16 pruebas evaluadas lograron discriminar los tres niveles de competencia, y 15 pruebas lograron discriminar al menos dos de los tres grupos (α= 0.05). Adicionalmente, otras dos pruebas diferenciaron los novatos de los residentes, y otras siete pruebas diferenciaron los residentes de los expertos.

**Conclusión:**

El nivel de competencia de un practicante de cirugía mínimamente invasiva puede ser determinado mediante una colección de pruebas básicas en un simulador quirúrgico físico. El diseño de pruebas que discriminen los tres niveles de competencia y reduzcan el número de pruebas de la colección son posibles temas para nuevos trabajos.

## Introduction

Minimally invasive surgery (MIS) allows procedures without big incisions, decreasing the risk of lesions, hemorrhages and the time of post operatory recovery 1. However, this surgical technique requires watching the surgical place throughout a screen and to move the hands out of surgeon´s view, limiting his/her visual field and modifying of his/her visual depth and force feedback 2. These problems demand from surgeon a competency that is solely acquired through practice, since the needed skills to operate in such conditions are not intuitive, common during daily live activities or required during open surgery 3.

Research and medical community have aimed at developing new and most appropriate techniques for training and evaluation of new surgeons 4-7. Regional references on the topic, to the knowledge of the authors of this study, are almost inexistent 8. Education, evaluation and certification in surgery are mostly determined by the relationship between master and disciple, in which a surgery resident must assist a senior surgeon during surgical duties, e.g. a MIS technique, and follow his recommendations until the senior surgeon considers the resident is ready to perform the duty without supervision. Particularly, the evaluation of surgery residents is based on the memory of the senior surgeon, and therefore, it is subjective and prone to err 3. Popular evaluation methodologies include the "Objective Structured Assesment of Technical Skills" (OSATS) 9 and observational tools for assessment of procedural skills. However, evaluation of technical skills using current observational assessment tools is not reliable and valid for all the competency levels 10. 

Several available simulators are used to teach and train the basic and necessary skills needed in MIS 11. The practice with virtual reality (VR) simulators allows the surgeon to experiment conditions similar to the ones found in a real surgery, but without the risk and cost of real surgery. Other type of simulator are the training boxes, mock-up models that allow to practice in a tridimensional space with a more realistic visual and tactile feedbacks but a lower price than the one of using VR simulators 12. However, training boxes are often an oversimplification of the patient and the evaluation performed with them is also commonly subjective 13.

In general, teaching MIS skills most include objective evaluations that allow assessing and certifying the learning curve and level of expertise of the MIS practitioners. Discrimination of different levels of competency in MIS is, therefore, a basic requirement in an evaluation method for MIS practitioners. This study aims at evaluating if a collection of simple tests in a trainer box is able to assess and classify correctly a group of MIS practitioners into three groups accordingly with their competency level: novice, resident and expert surgeon. 

## Materials and Methods

The methodology in this study includes three sequential parts: definition of the collection tests, an experiment, and an analysis of the results. 

### Definition of the collection tests

**Figure 1.**
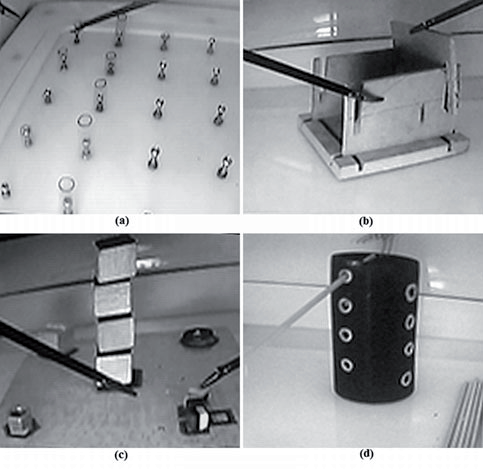
Displacement tests: (a) De1-Cylinders: to order ten cylinders from the smallest to the largest in a board, (b) De2-Boxes: to build two boxes using five wooden components, (c) De3-Tower: to build up a tower made of four cubes and to locate four objects around the tower, and (d) De4-Sticks: to cross a hollow cylinder with eight sticks throughout some predefined holes.

Several different tests were studied and modified from previous literature 8,14 by an interdisciplinary team including a senior surgeon. In total 16 tests were implemented for this study. The aim of each test was to evaluate one of four fundamental skills in MIS: displacement (De), cut (C), dissection (Di), and suture (S). Each fundamental skill was evaluated by four tests. Criteria such as objectivity, economy and simplicity were taken into account at the moment of selecting the tests. The chosen tests are described as follows:

**Displacement tests **(Fig. 1). 

De1- "Cylinders": to order ten cylinders from the smallest to the largest in a board.

De2-"Boxes": to build two boxes using five wooden components.

De3-"Tower": To build up a tower made of four cubes and to locate four objects around the tower.

De4-"Sticks": To cross a hollow cylinder with eight sticks throughout some predefined holes. 

**Figure 2.**
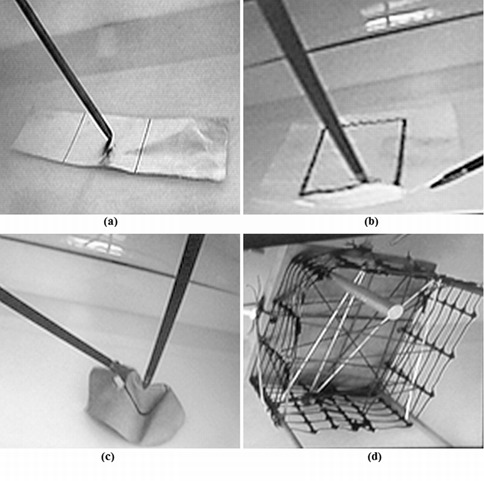
Cutting tests: (a) C1-Aluminum: to cut three squares following a pre-established path in aluminum fail, (b) C2-Figure: to cut a rectangle following a pre-established path in a sheet of paper, (c) C3-Half-balloon: to cut a circle following a pre-established path in an elastic balloon, and (d) C4-Mesh: cut the white threads from a mesh made of different color threads.

**Cutting tests **(Fig. 2).

C1- "Aluminum": To cut three squares following pre-established path in aluminum fail.

C2- "Figure": To cut a rectangle following pre-established path in a sheet of paper. 

C3- "Half-balloon": To cut a circle following pre-established path in an elastic balloon. 

C4- "Mesh": To cut the white threads from a mesh made of different color threads.

**Figure 3.**
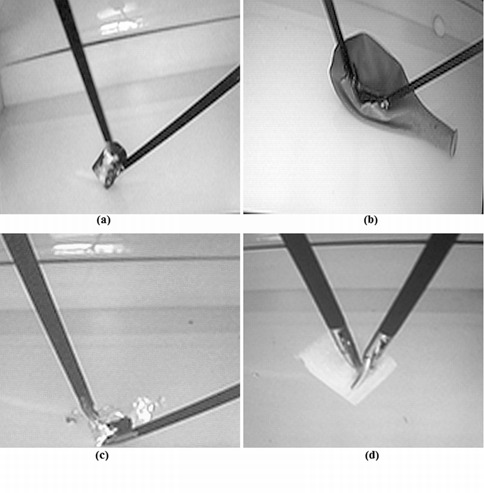
Dissection tests: (a) Di1-Plastic: to remove the plastic foil that covers a Play-Doh ball without scratching it, (b) Di2-Surgery: to remove three plastic spheres from an elastic balloon throughout a pre-established cutting path, (c) Di3-Glass: to remove an aluminum foil from around a glass ball without breaking the foil., and (d) Di4-Foam: to remove a foam-block glued to a surface without damaging the foam.

**Dissection tests **(Fig. 3).

Di1-"Plastic": To remove the plastic foil that covers a Play-Doh ball without scratching it.

Di2-"Surgery": To remove three plastic spheres from an elastic balloon throughout a pre-established cutting path. 

Di3-"Glass ball": To remove an aluminum foil from around a glass ball without breaking the foil.

Di4-"Foam": To remove a foam-block glued to a surface without damaging the foam.

**Figure 4.**
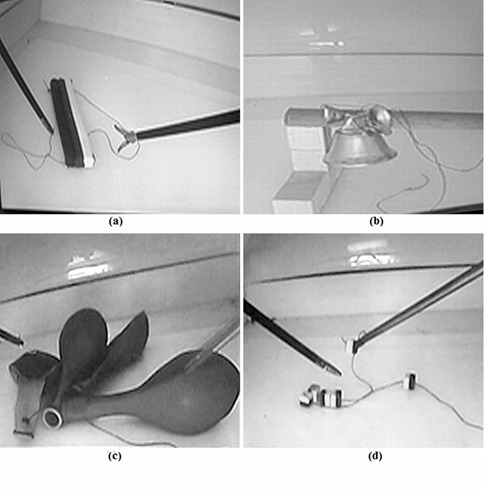
Suturing tests: (a) S1-Play-Doh: to suture three Play-Doh bars using a single thread, (b) S2-Bells: to tie up three toy bells to a stick using the rings attached to them, (c) S3-Ballons: to tie up four elastic balloons from their marked end, and (d) S4-Collar: to make a collar using six plastic rings and tie them up with a thread.

**Suturing tests **(Fig. 4).

S1-"Play-Doh": To suture three Play-Doh bars using a single thread.

S2- "Bells": To tie up three toy bells to a stick using the rings attached to them. 

S3- "Balloons": To tie up four elastic balloons from their marked end.

S4- "Collar": To make a collar using six plastic rings and tie them up with a thread.

Each one of the tests was evaluated by some characteristic indices as follows: Displacement tests were evaluated by accomplishment time, number of drops, and the release precision. Cutting tests were evaluated by accomplishment time, cut threads, length and precision of the cut. Suturing tests were evaluated by accomplishment time, number of fixed knots, and number of object tied up. Lastly, dissection tests were evaluated by accomplishment time, number of scratches to the target, and number of released spheres. Each the test had to be accomplished in less than five minutes. 

### Experiment

The hypothesis of this study, if a collection of simple tests in a trainer box is able to discriminate MIS practitioners into three categories (novices, residents and expert surgeons), was tested using an experimental design. 

Three groups of MIS practitioners were volunteers in this study. The first group, Novice group (A), was made of six students of our Medical School without any experience in surgery. The second group, Residents group (R), was made of six physicians in their final years of residency in surgery. The third and last group, Experts group (E), was made of six senior surgeon with recognized experience in MIS procedures. All the voluntaries (18 in total) were recruited at the Medical School in the Pontificia Universidad Javeriana (Bogotá, Colombia) and signed an informed consent form which indicated the risk of participating in the study (i.e. minor risk), the right to leave the study at any moment, and the privacy treatment of personal and collected data.

In a random order, the total of the 18 volunteers performed the simples tests described above. The order of the tests execution was also random. Before execution of each test, voluntaries watched a video clip in which a surgeon described the test through a demonstration and how it is evaluated. There was a single evaluator for each type of fundamental skill to be evaluated (displacement, cutting, suturing, and dissection). The evaluation of each test was done immediately after it was completed. The complete experiment was accomplished in five days. 

### Statistical analysis

To facilitate the analysis of the results, all the evaluation indicators were weighted accordingly to the performance of the volunteers as follows: R_tin_= BS_in_-S_tin_/BS_in_-WS_in_

were *R* *_tin_* is the relative performance for a volunteer *t *in the evaluation index *n* in the test *i, BS* *_in_* and *WS* *_in_* are the best and worst score for the evaluation index *n *obtained by any of the participants in the test, and *S* *_in_* is the score done by the participant *t* in the corresponding test and evaluation index. 

Additionally, a single grading index per participant Ṝ*_ti_* was calculated as the average of the relative performance of all the indices for a single test *i.*

An experimental design of a single factor (expertise or competency level) with three treatments (novice, resident, and expert) was used in this study. Therefore, an analysis of variance (ANOVA) was performed for the results of each simple test. A Statistical significance of 0.05 was selected to test the hypothesis, i.e. there is a significant effect of the group of the participant (competency level) on his/her results of the each test (Ṝ*_ti_*). Due to the significant results of ANOVA a Knewman-Keuls post-test was applied to the data to discover which groups of the three groups were significantly discriminate by the simple test. This statistical analysis was performed using Excel 2010^&reg;^ (Microsoft, USA). 

##  Results

**Figure 5.**
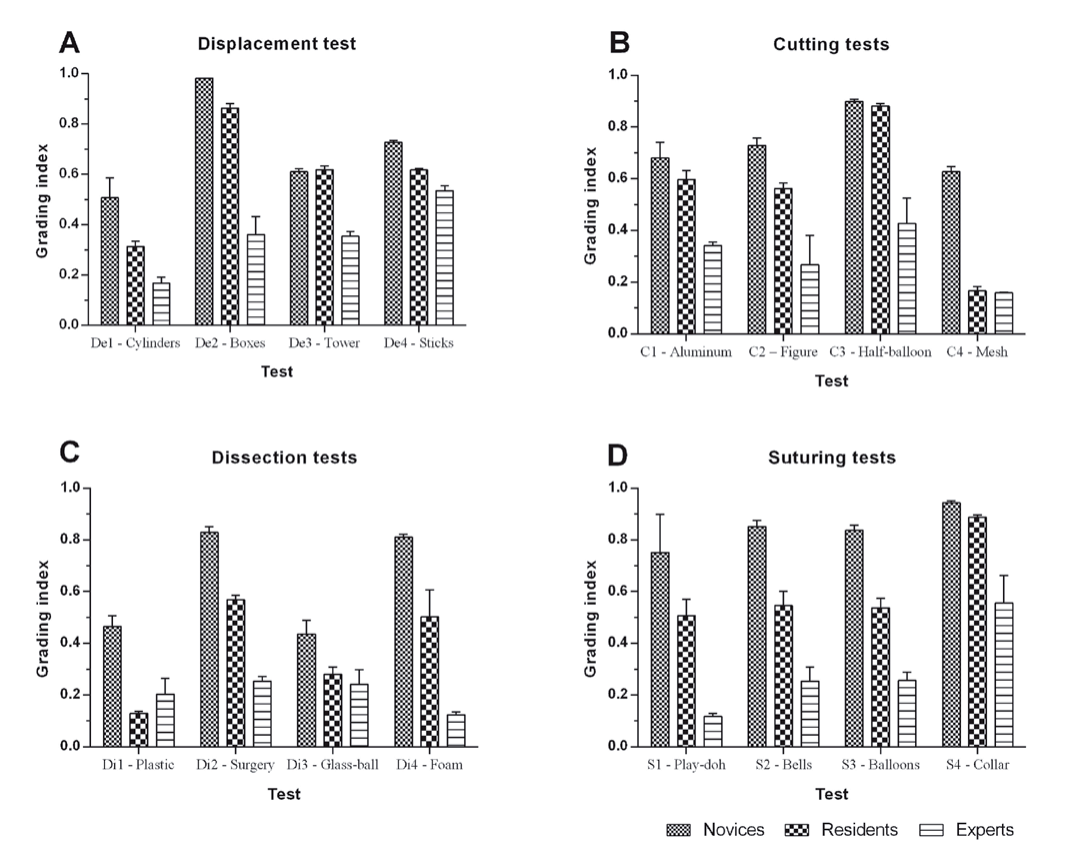
Dynamite plot for mean and standard deviation of Rti values for all tests and groups in tests of (A) displacement, (B) cutting, (C) dissection, and (D) suturing.

The average and standard deviation of the grading indices (Ṝ*_ti_*) per group are shown in Figure 5. The Experts group (E) obtained a better average score in all the tests, except in one dissection test (Di1-"Plastic") and the Residents group (R) obtained a better average scores than the Novices group (A), except in the displacement test De3-"Tower". For all the other tests the average score gave an adequate indicator of the competency level of the groups.

The ANOVA results pointed out that all the simple tests were able to discriminate at least one the study groups, except for the test Di3-"Glass ball" (*p* >0.05). The Student Newman Keuls (SNK) post-test analysis concluded that only four tests were able to discriminate correctly the three study groups, those tests were: Di2-"Surgury", Di4-"Foam" (both dissection tests), S2-"Bells", and S3-"Balloon" (both suturing tests). None of the displacement and cutting tests were able to discriminate all the study groups. The complete results for the SNK post-test can be seen in Table 1.

**Table 1.** Discrimination power of the different tests. Results of the Student Newman Keuls (SNK) post-test (α= 0.05).

Description	Grouping by SNK test*		Tests Means											
			S1	S2	S3	S4	C1	C2	C3	C4	Di1	Di2	De3	Di4
Tests able to discriminate all three groups	A	Novices		0.852	0.838							0.830		0.810
	B	Residents		0.546	0.537							0.569		0.503
	C	Experts		0.254	0.256							0.254		0.124
Tests that discriminate two groups where Novices and Residents are not significantly different from each other, and experts have a significant best performance than them.	A	Novices	0.751			0.944	0.680	0.728	0.899			0.982	0.610	
		Residents	0.507			0.889	0.596	0.562	0.881			0.863	0.619	
	B	Experts	0.117			0.556	0.341	0.267	0.427			0.361	0.355	
Novices are in a different group than Residents and Experts (that are in one cluster)	A	Novices								0.627	0.465			
	B	Residents								0.166	0.130			
		Experts								0.159	0.204			
Novices clearly differentiated from experts but Residents performance is not significantly different from novices nor from experts.	A	Novices									0.507			
	A	Residents									0.314			
	B	Experts									0.168			
Tests are not able to discriminate between any groups, i.e., Novices, Residents and Experts performances are not significantly different between them	A	Novices											0.436	
		Residents											0.280	
		Experts											0.241	

*with 5% of confidence

S: suture

C: cut

Di: dissection

Finally, several tests discriminated exclusively the groups Novices and Residents (C4-"Mesh", and Di1-"Plastic") and the groups Residents and Experts (De2-"Boxes, De3-"Tower", C1-"Aluminum", C2-"Figure", C3-"Half-balloon, S1-"Play-Doh" and S4-"Collar").

## Discussion

The advantages of box trainers over virtual reality simulators include real tactile feedback and accurate deepness reduction. However, the oversimplification of the represented activities, the absence of objective evaluation 13, and the lack of evidence for all sources of validity are the most relevant drawbacks that must be overcome in the future 7. The main objective of this study is to determine if a collection of simple tests in a box trainer can be used to discriminate, at least, three level of competency in MIS practitioners. We assume that discrimination between competency levels is a necessary and key step towards the design of an objective evaluation test of practitioners, and which inexistence is a major drawback of box trainers.

This study proposed 16 tests within box trainers that involved fundamental MIS tasks such as displacements, cutting, suturing, and dissection. However, not all the tests discriminated the level of competency of the tested practitioners. Only four designed tests of the entire collection were able to discriminate the three groups of study (Experts, Residents, and Novices). Therefore, these four tests (Di2-"Surgery", Di4-"Foam", S2-"Bells" and S3-"Balloons") can be considered as ideal as part of an evaluation of MIS practitioners. Similar results have been previously reported. A study 14 reported a similar number of tests involving displacements and cutting targets in a box trainer that successfully discriminate basic competency levels only among residents and classify correctly 74% of the tested practitioners using the OSATS. Using the same simulator, Emper *et al*. 15, discriminated only two basic levels of competency (novice vs. expert) using objective kinematics parameters such as path length, speed, and "smoothness" of the instruments motion. Fraser *et al*. 16, also succeed discriminating two levels of psychomotor skills (students and experts) using a collection of tests including displacements and cutting of targets and knots making. Fraser used a basic trainer box, while both Chmarra and Empel works used a box trainer with motion tracking system 14.

 This study also found that other nine tests of the collection of 16 were able to discriminate significantly the groups Novices and Residents, or the groups Residents-Experts, and therefore, they could complement the previous four tests that effectively discriminated all the three studied groups. For instance, combined two of these nine tests correctly assessed and classified the studied groups in tasks of displacement, a task not evaluated by the three tests previously mentioned. The nine tests were C4-"Mesh", Di1-"Plastic", De2-"Boxes", De3-"Tower", C1-"Aluminum", C2-"Figure", C3-"Half-balloon", S1-"Play-Doh" and S4-"Collar".

 However, the usefulness of the mentioned seven tests is limited to their use as part of a collection of tests. Their use as independent tests can be source of error in a competency assessment. For instance, the test Di1-"Plastic" differentiated -not in a significant fashion- the groups Residents and Experts but the grading indices was incorrectly better for the Residents group. A similar problem was seen with the test De3-"Tower". 

A main limitation of this study is that the definition of all the characteristic indices for the test might be cumbersome. For instance, evaluation of the "precision" in a cutting task was assessed by measuring the maximum deviation of the cut from the pre-established guiding path. However, selection of the maximum deviation point and measuring with a common metric ruler was ungainly and it did not added objectivity to the test. A re-definition of the indices to make them easier to work with them and ensure objectivity may a future work. 

## Conclusion

The proposed collection of simple tests in a trainer box was able to assess and classify correctly three groups of MIS practitioners accordingly with their competency level: novice, resident and expert surgeons. However, this result relies more in some of the proposed testes than the others. This is, only four of the tests were able to classify correctly the MIS practitioner into the three study groups. However, those four tests assessed the practitioners in only two of the four identified basic MIS tasks (dissection and suturing). There is still a need of both designing simple tests in tasks of displacement and cutting that can complement a basic evaluation of psychomotor evaluation of the MIS practitioners, and the redefinition of some evaluation criteria to improve the overall objectivity of the evaluation. Further research is necessary for the development of evaluation and training of MIS surgeons, particularly at regional level. 
